# Opposite effect of basic combat training on mood state of recruits with different physical fitness: A study from perspective of fatigue

**DOI:** 10.3389/fpsyg.2022.961351

**Published:** 2022-09-09

**Authors:** Yi Ruan, Shang-jin Song, Zi-fei Yin, Xin Wang, Bin Zou, Huan Wang, Wei Gu, Chang-quan Ling

**Affiliations:** ^1^School of Traditional Chinese Medicine, Naval Medical University, Shanghai, China; ^2^Department of Traditional Chinese Medicine, Xingcheng Sanatorium of PLA Strategic Support Force, Xingcheng, Liaoning, China

**Keywords:** mood state, basic combat training, borg rating of perceived exertion scale, profile of mood state questionnaire, soldier

## Abstract

**Objective:**

Basic combat training (BCT) is a kind of necessary high-intensity training to help each military recruit convert into a qualified soldier. In China, both the physical fatigue and passive psychological state have been observed in new recruits during BCT. However, after same-intensity training, the degree of fatigue and passive mood vary among recruits. Therefore, this study aimed to explore the effect of BCT on mood state of recruits with different physical fitness levels from a perspective of fatigue.

**Materials and methods:**

Before and after BCT, the degree of fatigue and mood state of participants were evaluated via the Borg Rating of Perceived Exertion Scale and Profile of Mood States Questionnaire immediately after 20 push-ups as RPE and POMS scores [total mood disturbance (TMD), passive mood (T_tension_, T_anger_, T_fatigue_, T_depression_, and T_confusion_) and positive mood (T_vigour_ and T_esteem_)]. The participants were divided into two groups according to the RPE score measured after BCT: (1) group 1: RPE score after BCT < 13 and (2) group 2: RPE score after BCT ≥ 13.

**Result:**

A total of 564 recruits were included (group 1: 456/564, 80.85%; group 2: 108/564, 19.15%). After BCT, in group 1, TMD (from 95.65 ± 17.89 to 87.52 ± 17.63) and passive mood T_tension_ (from 4.46 ± 3.18 to 3.79 ± 3.14), T_fatigue_ (from 4.94 ± 3.58 to 3.12 ± 3.04), T_depression_ (from 2.86 ± 3.41 to 2.01 ± 2.75), T_confusion_ (3.12 ± 2.72 to 2.42 ± 2.57) declined significantly (all within-group *p* < 0.001), but positive mood both increased significantly (T_vigour_: from 13.21 ± 4.59 to 15.44 ± 5.42, T_esteem:_ from 9.18 ± 3.36 to 11.04 ± 3.67; both within-group *p* < 0.001); while in group 2, only T_anger_ (from 4.27 ± 4.16 to 6.22 ± 5.94, within-group *p* = 0.001) and T_esteem_ (from 8.36 ± 3.15 to 9.07 ± 3.67, within-group *p* = 0.031) increased significantly.

**Conclusion:**

BCT could alleviate passive mood and add to positive mood for recruits with better physical fitness, while had no ameliorative effects on or even deteriorate most of the passive mood for recruits with worse physical fitness.

## Introduction

In China, basic combat training (BCT) is a stage of military training designed to help a recruit convert from an ordinary citizen into a qualified soldier via high-intensity and high-quality military training (Gao et al., 2012). Although the specific training items have some alternations among different military occupational specialties, all recruits in any of the specialties are forced to undergo BCT, which usually covering unified basic physical training and specific tactical training, and lasts for 10 to 14 weeks (Ruan et al., 2021).

Previous study showed that physical inactivity has proven resistant to be trained and the decision to engage in exercise is based on psychological factors (Ekkekakis et al., 2011). Therefore, mood state, the main manifestation of psychological state, becomes an important focus of training and exercise, especially military training (Clemente-Suarez et al., 2018). However, the effects of military training on mood state varies from study to study. Lieberman et al. reported that 9- to 10-week United States army BCT could improve the mood state of female recruits (Lieberman et al., 2014) as well as male recruits (Lieberman et al., 2016). While Shannon et al. reported 14.3% recruits suffered from depressive symptoms after an 8-week Columbian army BCT (Crowley et al., 2015). In China, the status is similar: both positive and passive psychological state have been observed in new recruits during BCT (Wang, 2007; Wu et al., 2015).

Ekkekakis et al. reviewed 33 articles on the relationship between the intensity of non-military exercise and affective responses, and concluded that pleasure feeling is often reduced when the exercise is intensive (Ekkekakis et al., 2011). While, study also shows that the psychophysiological response to BCT may be related to recruits’ inadaptation to new environments full of physical training and daily schedules which are in stark contrast to their prior living environments (Crowley et al., 2015).

Actually, though fatigue is one of our body’s normal physical reactions to intensive training, however, as reported by previous study of Chinese BCT, even under the same-intensity training, recruits often have different degrees of fatigue (Gao et al., 2012), implying the existing difference of individual physical fitness within the same batch of recruits.

Many studies are exploring the risk factors of training fatigue and passive psychological state separately (Crews, 1992; Härmä et al., 2019), yet none has directly addressed the effect of BCT on psychological state among recruits with different degrees of fatigue.

Up to this point, this study aimed to explore the potential differential effect of BCT on mood state among recruits with different physical fitness levels measured as degree of self-reported fatigue in a Chinese new recruit population in 2018: we hypothesized that when conducting a specific type of intensity training, new recruits with better physical fitness levels would report lower degrees of fatigue and those with worse physical fitness levels would report higher degrees of fatigue.

## Materials and methods

### Study overview and ethical approval

From the end of September 2018 to December 2018, we followed a batch of male recruits undergoing BCT at a basic training base in China. This BCT is a 12-week high-intensity military training course covering basic physical and tactical training, according the requirements of the Chinese Military Training Programme.

Baseline assessment survey included the collection of demographic characteristics and a detailed explanation of the study by research staff from the School of Traditional Chinese Medicine and the First Affiliation Hospital of Naval Medical University, Shanghai, China. Recruits were informed that participation in the study was voluntary, and they signed a written informed consent.

A total of 2 assessment surveys were conducted: before (the end of September 2018) and after (the end of December 2018) the BCT, we surveyed the degree of fatigue and mood state of participants (for details see Parts 2.3 and 2.4 below). The degree of fatigue and mood state were quantified through the Borg Rating of Perceived Exertion Scale and the Profile of Mood States (POMS) Questionnaire, respectively. The recruits were under a unified military command imposing unified training, daily routine schedule and dietary intake.

This study was approved by the Shanghai Changhai Hospital Ethics Committee (No: 2018-048), and performed in accordance with the Declaration of Helsinki.

### Selection of participants

Participants were selected via cluster sampling: first 2 battalions (n = 798) were randomly selected from all battalions undergoing navy BCT; then, individual recruits were interviewed for willingness to participate in this study.

The inclusion criteria for participants were: (1) qualified recruits in the training base where the study is being conducted in 2018; (2) without major organic diseases or limb joint damage that could prevent participation in daily military training tasks; (3) willing to participate in this study and promised the authenticity of the subjective survey questions; and (4) signing the informed consent.

The exclusion criteria of participants were: (1) having taken one or more drugs that may cause fatigue and mood fluctuation within the 2 weeks prior to BCT and (2) suffering from diseases that may cause fatigue and mood fluctuation, such as colds, fibromyalgia, depression, tristimania, etc., within the 2 weeks prior to BCT.

Subjects who participated in this study had the right to withdraw from the study at any time without reason. The subjects who had not explicitly proposed to withdraw from the study but were absent for one or both surveys (before and after BCT) were regarded as withdrawn. The data of withdrawn participants were excluded from data analysis.

### Fatigue measurement and group definition

The physical fitness was reported as degree of self-reported fatigue measured by the Borg Rating of Perceived Exertion Scale, marked as RPE value. The baseline and post-BCT RPE values were determined one day before and after BCT, respectively.

The Borg Rating of Perceived Exertion Scale is a widely used psycho-physical tool to semi-quantitatively assess subjective perception of effort during exercise (Feng et al., 2003). Studies have shown that this scale has good evaluation efficacy, and the RPE value is closely related to exercise load intensity, heart rate, oxygen consumption, lactic acid, and hormones (Aamot et al., 2014). This scale has 15 rates in total, from 6 to 20, with a higher rate score indicating a higher degree of perceived fatigue. Generally, individuals with RPE scores ≥ 13 can be regarded as suffering from exercise-induced fatigue (Ruan, 2019).

When determining RPE values, participants were first informed about the instructions of the Borg Rating of Perceived Exertion Scale as follows: “You will do 20 push-ups in 1 min. Immediately after the push-ups, we want you to rate your perception of exertion as how heavy and strenuous this exercise feels to you and how tired you are. The perception of exertion is mainly felt as strain and fatigue in your muscles and as breathlessness or aches in the chest as explained by this card”. Then, every 20 participants were examined simultaneously. A card explaining the meaning of each rate of the Borg’s Rating of Perceived Exertion Scale was put before each participant. Participants were required to do 20 push-ups under the command of drill sergeants. Finally, each participant was required to report on his degree of fatigue as in the above instructions. For each participant, one drill sergeant was assigned to record the RPE value reported by participants immediately after the 20th push-up.

To further explore the changing characteristics of the mood state of subjects with different levels of fatigue after BCT, the participants were divided into a non-fatigue group and a fatigue group according to the RPE score measured after BCT: (1) group 1 (non-fatigue group, participants having average or good physical fitness): RPE score after BCT < 13 and (2) group 2 (fatigue group, participants having low physical fitness): RPE score after BCT ≥ 13.

### Mood state measurement

The mood state was measured by the Profiles Mood State (POMS) questionnaire. The POMS is a standard validated psychological test used in research for evaluation of mood state (McNair et al., 1971).

We used the Chinese form of the POMS Questionnaire designed for adult athletes with good reliability (Cronbach α = 0.71) (Zhu, 1995), which comprised 40 evenly distributed items in 7 first-order factors: tension (items 1, 8, 15, 21, 28, and 35), anger (items 2, 9, 16, 22, 29, 36, and 37), fatigue (items 3, 10, 17, 23, and 30), depression (items 4, 11, 18, 24, 31, and 38), vigor (items 5, 12, 19, 25, 32, and 39), confusion (items 6, 13, 20, 26, and 33), and esteem (items 7, 14, 27, 34, and 40). Each item was rated in five-ordered categories that received scores between 0 (not at all) and 4 (extremely). The total score of each one of the 7 factors was the sum score of items under this factor, marked as T_tension_, T_anger_, T_fatigue_, T_depression_, T_vigour_, T_confusion_, and T_esteem_. The total mood disturbance (TMD) was calculated as the score of passive mood (tension, anger, fatigue, depression, vigor, and confusion) minus those of positive mood (vigor and esteem) as follows: TMD = T_tension_ + T_anger_ + T_fatigue_ + T_depression_ – T_vigour_ + T_confusion_ – T_esteem_. For each factor, a higher score indicated a higher degree of corresponding mood state, and a higher TMD indicated a higher degree of mood disturbance.

The baseline and post-BCT POMS questionnaire were administered to participants one day before and after BCT, respectively, after the RPE score determination. When the POMS questionnaire was surveyed, participants were first required to sit in the auditorium with one seat empty between adjacent participants. Then, two researchers handed out the POMS Questionnaire to each participant. The participants were required to answer the items on the questionnaire one by one according to the questionnaire’s instructions written before the first item on the questionnaire. If anyone had any questions about the meaning of items on the POMS Questionnaire, he could raise his hand and ask for the assistance of researchers.

Any POMS Questionnaire with at least one blank item was ruled out from data analysis.

### Statistical analysis

Statistical analysis was performed using IBM SPSS Statistics version 21.0 (IBM Corp., Armonk, NY, United States). Measurement data were expressed as the mean ± standard deviation; within-group comparisons were performed using the paired *t*-test or Wilcoxon signed rank test for normality and non-normality distributed data, respectively; and between-group differences were compared using Student’s *t*-test or Mann-Whitney U test, respectively. The normality of measurement data was tested via Kolmogorov-Smirnov test. Categorical data were expressed as frequency and percentage (%), and between-group comparisons were performed using the chi-squared test. A *P* < 0.05 was considered statistically significant.

## Results

### Demographic characteristics of recruits

**FIGURE 1:**
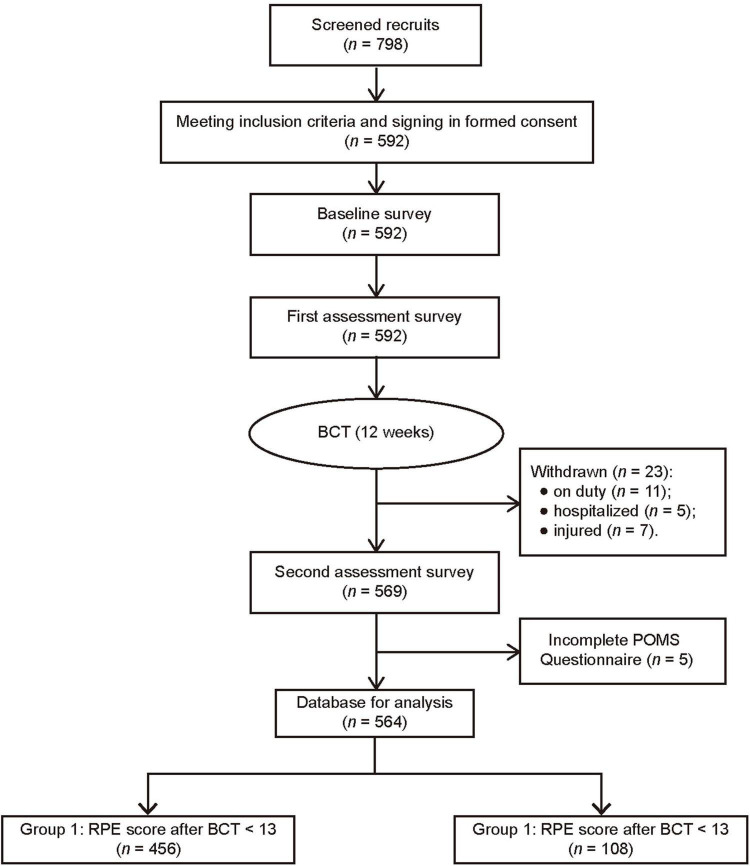
Flow chart of study.

A total of 798 recruits were screened, and only 592 recruits participated in this study at baseline survey. After BCT, 23 participants were withdrawn from this study for the following reasons: on duty (*n* = 11), hospitalized (*n* = 5), and injured (cannot finish 20 push-ups, *n* = 7). Among the 569 participants who finished the post-BCT assessment, the data of 5 were deleted from analysis due to an incomplete POMS questionnaire. Therefore, only the data of 564 participants were finally analyzed (Figure 1).

As shown in Table 1, among the 564 participants, after BCT, 456 did not feel fatigue after conducting 20 push-ups within 1 min (group 1), and 108 recruits could not finish 20 push-ups within 1 min with ease (group 2). In addition, the mean age of the participants was comparable between groups (20.27 ± 1.39 vs. 20.26 ± 1.59 years for group 1 and group 2, Z = –0.597, *p* = 0.551). The majority were of Han ethnicity (98.7% and 98.1% in group 1 and group 2, χ^2^ = 0.179, *p* = 0.653) and had an education level of an associate’s degree or some college (64.3% and 69.4% in group 1 and group 2, χ^2^ = 1.666, *p* = 0.435). The majority families of the participants could make both ends meet (74.3% and 66.7% in group 1 and group 2, χ^2^ = 7.049, *p* = 0.070). All these demographic characteristics had no significant differences between groups 1 and 2 (all *p* > 0.05).

**TABLE 1:** Demographic characteristics and Borg Rating of Perceived Exertion scale (RPE) score of participants (*N* = 564).

Characteristics	Group 1	Group 2	Statistics ^#^	*p*-value
Participants (*n* [%])	456 (80.85%)	108 (19.15%)	–	–
Age (years, mean ± SD)	20.27 ± 1.39	20.26 ± 1.59	–0.597	0.551
Ethnicity (*n* [%])			0.179	0.653
Han	450 (98.7%)	106 (98.1%)		
Non-Han	6 (1.3%)	2 (1.9%)		
Education (*n* [%])			1.666	0.435
High school degree	111 (24.3%)	20 (18.5%)		
Associate’s degree or some college	293 (64.3%)	75 (69.4%)		
College degree or higher	52 (11.4%)	13 (12.0%)		
Family income (*n* [%])			7.049	0.070
Considerable debt	13 (2.9%)	2 (1.9%)		
Slight debt	96 (21.1%)	34 (31.5%)		
Make both ends meet	339 (74.3%)	72 (66.7%)		
Rich	8 (1.8%)	0 (0.0%)		
RPE score (mean ± SD)				
Before BCT	10.69 ± 2.30	11.95 ± 2.67	5.040	< 0.001
After BCT	10.28 ± 1.67	13.94 ± 1.41	16.463	< 0.001
Change before and after BCT	↓*	↑*		

Group 1 (non-fatigue group): RPE after BCT < 13; group 2 (fatigue group): RPE after BCT ≥ 13. Between-group *p* value of measurement data was calculated by Mann-Whitney U test since Kolmogorov-Smirnov test verified they were non-normality data, and *p* value of categorical data was calculated by chi-squared test. ^#^ Statistics were shown as χ^2^ for Chi-square test, and Z for Mann-Whitney U test. *: within-group p-value was calculated by Wilcoxon signed rank test as 0.001 (Z = –3.188) and < 0.001 (Z = 6.369) for group 1 and group 2, respectively. ↑: increased significantly after BCT; ↓: decreased significantly after BCT. BCT, basic combat training; RPE, score of the Borg Rating of Perceived Exertion scale; SD, standard deviation.

### Borg rating of perceived exertion scale score of recruits before and after basic combat training

For the RPE score of recruits before and after BCT, as shown in Table 1, after BCT, the RPE scores after 20 push-ups were significantly decreased in group 1 (from 10.69 ± 2.30 to 10.28 ± 1.67, *Z* = –3.188, within-group *p* = 0.001), but significantly increased in group 2 (from 11.95 ± 2.67 to 13.94 ± 1.41, *Z* = 6.369, within-group *p* < 0.001). The RPE scores of group 1 were significantly lower than group 2 both before (10.69 ± 2.30 vs. 11.95 ± 2.67, *Z* = 5.040, between-group *p* < 0.001) and after BCT (10.28 ± 1.67 vs. 13.94 ± 1.41, *Z* = 16.463, between-group *p* < 0.001).

### POMS score before and after combat training

The POMS score of group 1 (Table 2, group 1) showed that, after BCT, the T scores of passive mood, except anger (from 2.66 ± 2.98 to 2.67 ± 3.07, *Z* = 0.466, within-group *p* = 0.641), all declined significantly (within-group *p* < 0.001), including tension (from 4.46 ± 3.18 to 3.79 ± 3.14, *Z* = –4.312), fatigue (from 4.94 ± 3.58 to 3.12 ± 3.04, *Z* = –4.312), depression, and confusion. The TMD score also declined significantly (within-group *p* < 0.001). However, the T scores of positive mood, vigor (from 13.21 ± 4.59 to 15.44 ± 5.42, *Z* = 9.344) and esteem (from 9.18 ± 3.36 to 11.04 ± 3.67, *Z* = 10.710), both increased significantly (within-group *p* < 0.001).

**TABLE 2:** Profile of Mood States (POMS) score of recruits in 2 groups.

Item	Group 1 (*n* = 456)					Group 2 (*n* = 108)				
	Before BCT	After BCT	Change	Z	Within-group *p*-value	Before BCT	After BCT	Change	Z	Within-group *p*-value
T_tension_	4.46 ± 3.18	3.79 ± 3.14	↓	–4.312	< 0.001	6.52 ± 4.06	7.08 ± 4.31	–	1.491	0.136
T_anger_	2.66 ± 2.98	2.67 ± 3.07	–	0.466	0.641	4.27 ± 4.16	6.22 ± 5.94	↑	3.327	0.001
T_fatigue_	4.94 ± 3.58	3.12 ± 3.04	↓	–10.232	< 0.001	7.38 ± 4.34	7.74 ± 5.00	–	0.730	0.465
T_depression_	2.86 ± 3.41	2.01 ± 2.75	↓	–5.461	< 0.001	4.92 ± 4.18	5.12 ± 5.28	–	0.016	0.987
T_vigour_*	13.21 ± 4.59	15.44 ± 5.42	↑	9.344	< 0.001	11.09 ± 4.68	11.44 ± 5.10	–	0.861	0.389
T_confusion_	3.12 ± 2.72	2.42 ± 2.57	↓	–5.623	< 0.001	4.84 ± 2.89	4.77 ± 3.89	–	–0.734	0.463
T_esteem_*	9.18 ± 3.36	11.04 ± 3.67	↑	10.710	< 0.001	8.36 ± 3.15	9.07 ± 3.67	↑	2.156	0.031
TMD	95.65 ± 17.89	87.52 ± 17.63	↓	–10.139	< 0.001	108.47 ± 21.88	110.42 ± 25.38	–	0.574	0.566

Group 1 (non-fatigue group): RPE after BCT < 13. Group 2 (fatigue group): RPE after BCT ≥ 13. Data were shown as the mean ± standard deviation. Within-group *P* value was calculated by Wilcoxon signed rank test. BCT: basic combat training; RPE: score of the Borg Rating of Perceived Exertion scale; POMS: Profile of Mood States Questionnaire. T_tension_, T_anger_, T_fatigue_, T_depression_, T_vigour_, T_confusion_, and T_esteem_ were the evaluation scores of 7 factors of POMS (T score). T score denoted with * were positive mood, others were negative mood. TMD: total mood disturbance evaluated by POMS. ↑: increased significantly after BCT; ↓: decreased significantly after BCT; –: no significant difference before and after BCT.

The POMS score of group 2 (Table 2, group 2) showed that, after BCT, the score of passive mood, T_anger_, increased significantly (from 4.27 ± 4.16 to 6.22 ± 5.94, *Z* = 3.327, within-group *p* = 0.001), but TMD and T score of other passive mood, including tension, fatigue, depression, and confusion, had no significant alternation (within-group *p* > 0.05). The score of positive mood, T_esteem_, increased significantly (from 8.36 ± 3.15 to 9.07 ± 3.67, Z = 2.156, within-group *p* = 0.031), but that of vigor had no significant alternation (within-group *p* = 0.389).

### POMS difference between groups

**FIGURE 2:**
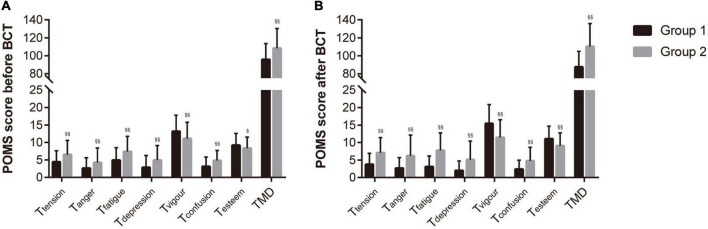
POMS score before **(A)** and after **(B)** BCT among group 1 (*n* = 456) and group 2 (*n* = 108). Data were shown as mean ± standard deviation. Between-group p-value was calculated by Mann-Whitney U test. § : *p* < 0.05 vs. group 1; §§ : *p* < 0.001 vs. group 1. BCT, basic combating training; POMS, Profile of Mood States Questionnaire. T_tension_, T_anger_, T_fatigue_, T_depression_, T_vigour_, T_confusion_, and T_esteem_ were the evaluation scores of 7 factors of POMS (T score). T score denoted with * were positive mood, others were passive mood. TMD, total mood disturbance evaluated by POMS.

As shown in Figure 2, both before and after BCT, the TMD and all the 5 T scores of passive mood (tension, anger, fatigue, confusion, and esteem) in group 1 were significantly lower than group 2 (all between-group *p* < 0.001); while all the 2 T scores of positive mood (vigor and esteem) were significantly higher in group 1 than group 2 (all between-group *p* < 0.05). The mean, standard deviation, effect size (Z), and exact *p*-value were reported in Supplementary Table 1.

## Discussion

### Summary of findings

Results of this study show that, after BCT, although the majority of recruits (group 1, 456/564, 80.85%) had enough physical fitness to finish 20 push-ups within 1 min, there were still some recruits (group 2, 108/564, 19.15%) who could not finish 20 push-ups with ease (RPE post push-up exercise after BCT > 13, Table 1).

The Borg Rating of Perceived Exertion Scale is a verified tool to evaluate exercise-induced fatigue as RPE score (Soriano-Maldonado et al., 2014). Generally, RPE < 9 indicted the feeling of very light exertion like a healthy person taking a short walk, RPE = 13 indicated the physical activity is somewhat hard for a person but to continue is no problem, and when RPE increases to 15, the person feels tiring and has difficulty to continue the activity (Borg, 1998). In our study, the RPE scores show that, from the perspective of fatigue, BCT can benefit recruits in group 1 (RPE post push-up exercise after BCT declined significantly, Table 2) but may have passive effects on recruits in group 2 (RPE post push-up exercise after BCT increased significantly, Table 1). This result not only indicates the individual distinguish of physical fitness among recruits but also implies that the existing recruit training schedule in the Chinese navy cannot fit for all recruits, especially for some recruits with worse physical fitness at baseline (like group 2). Although they would not present with intolerance of some military training items at the beginning (mean RPE post push-up exercise before BCT: 11.95 < 13, Table 1), the BCT actually deteriorated their physical fitness (mean RPE post push-up exercise increased significantly into 13.94 > 13, Table 1). Maybe the establishment of an individualized recruit training schedule can benefit more navy recruits in China.

Profile of mood states (POMS) questionnaire is widely used among military population to quantitatively evaluate the mood state. Previous studies observed decrease, increase, and no-change of POMS score (TMD and 7 factors T scores) among solders undergoing exercise or training (Lochbaum et al., 2021; Martínez-Díaz and Carrasco, 2021; Liao et al., 2022). However, the mood state discrepancy of Chinese recruits with different physical fitness after BCT was not explored before our study. For mood state evaluation, firstly, our results show that navy BCT had opposite effects on mood states among recruits with good or poor physical fitness. For recruits with better physical fitness (group 1), BCT can alleviate almost all passive mood (except anger) and mood disturbance (TMD) and add to positive mood (Table 2, group 1). However, for recruits with worse physical fitness (group 2), BCT not only had no ameliorative effects on most of the passive mood but also can even deteriorate passive mood, such as anger (Table 2, group 2). These results indicate that exercise/training, such as BCT, may be a mood regulator, but its effect does have individual differences depending on discrepancies in physical fitness, indicating that POMS score may be a potential indicator for fatigue assessment.

Besides, the group difference of POMS before and after BCT shows that recruits with better physical fitness (group 1) had better mood state, i.e., higher T scores of positive mood, and lower T scores of passive mood, than those with poorer physical fitness (group 2) at baseline (Figure 1A), and this kind of advance can only be intensified rather than be reversed after BCT (Figure 1B). These results contribute to the conclusion that BCT had opposite effects on mood states among recruits with good or poor physical fitness.

### Potential mechanisms

Exercise-induced fatigue is a kind of state covers the interaction of both physical fatigue manifesting as the descending physiological function of the body to maintain a predetermined exercise intensity (Hou et al., 2017), and psychological fatigue manifesting as the reluctance to be trained (Vrijkotte et al., 2018), this means that both the fatigue degree and psychological state of a person can be affected by a certain course of training. What is more, though fatigue may be inevitable under intensive exercise/training, individuals with different physical fitness could have varied response to a certain intensity training (Backhouse et al., 2007), leading to the distinguished results of our study.

The potential mechanisms behind the opposite effect of exercise/training on mood states with different degrees of fatigue were explored by both traditional Chinese medicine and Western medicine researchers, but the specific mechanism remains unclear.

In the theory of Western medicine, the benefit effects of exercise/training on mood states may be related to the activation of the hypothalamus-pituitary-adrenal axis: proper exercise training can increase the level of the glucocorticoid cortisol and dopamine in the prefrontal cortex of the medial brain, which can lead to positive emotions in exercised individuals (Chen et al., 2017). Besides, some studies reported that sleep loss (Lieberman et al., 2002), accumulation of lipid and plasma metabolites (Lieberman et al., 2012), and prolonged periods of cognitive workload (Zhang et al., 2021) could degrade mood state and trigger or add to the degree of fatigue. During intensive BCT, sleep loss is a common event which could lead to poor physical fitness (Lee and Lin, 2007), hinder the recovery and adaptive process from fatigue (Watson, 2017). Accumulation of lipid and plasma metabolites is a kind of results of muscular fatigue, which could trigger mental fatigue like passive mood state (Deely et al., 2022).

From the perspective of Chinese medicine theory (Wang et al., 2011; Yu et al., 2013), the liver not only governs the physical movement of the body, but also is the governor of the free flow of qi and blood in the body that can be a regulator for mood state. Therefore, moderate exercise or training can regulate the function of the liver, thereby regulating the qi and blood of the liver and finally regulating the mood state of the player.

### Strengths and limitations

This study discussed the opposite effects of BCT on mood states among recruits with average or low physical fitness from the perspective of fatigue assessment; therefore, it can be a reference for the psychological and fatigue management of recruits during BCT.

Also, this study also has a few limitations. First, it is only an observational research. Although our data supported the conclusion of this effect discrepancy of BCT on mood state among recruits with different degrees of physical fitness manifested as fatigue, suggesting the potential of POMS score as a fatigue indicator, more prospective studies with confirmative conclusions are required to verify our discovery. In addition, we only investigated recruits in the Chinese navy, thus more novel findings are needed to clarify the relationship between BCT and mood for other military occupational specialties in China or recruits in other countries around the world.

### Practical implications

The results of our study indicate that policies and practices encouraging individualized BCT schedule, and paying attention on the mood state of recruits with lower physical fitness might be beneficial in promoting the efficacy and safety. Also, moderate psychological assistance or intervention, and timely adjust of training intensity for specific recruits like those with lower physical fitness may reduce the degree of BCT-related fatigue, promote the effectiveness of BCT, and training adaptation.

## Conclusion

This study suggested that, from the perspective of fatigue assessment, BCT had opposite effects on the mood state of navy recruits with different degrees of physical fitness in China. BCT had beneficial effects on the mood state of recruits with average or good physical fitness but had no ameliorative effects on the majority of passive mood and even deteriorated some passive mood in recruits with poor physical fitness.

## Data availability statement

The original contributions presented in this study are included in the article/Supplementary material, further inquiries can be directed to the corresponding authors.

## Ethics statement

This study was approved by the Shanghai Changhai Hospital Ethics Committee (No: 2018-048). The patients/participants provided their written informed consent to participate in this study.

## Author contributions

WG and C-QL put forward the conception and design of the study. YR, S-JS, XW, BZ, and HW collected the original data. S-JS, YR, and Z-FY analyzed and interpreted the data. Z-FY, WG, and C-QL revised it critically. All authors drafted the article and approved the final version to be submitted.
