# Supplementary material for: Opposite effect of basic combat training on mood state of recruits with different physical fitness: A study from perspective of fatigue
Source: Front Psychol. 2022 Sep 9;13:961351. doi: 10.3389/fpsyg.2022.961351 (PMC9505696; doi:10.3389/fpsyg.2022.961351)
Supplement: Supplementary file 1 [file Table_1.docx]

Supplementary Material

# Table S1. Between-group difference of POMS score before and after BCT

| Item | Before BCT | | | | After BCT | | | |
| --- | --- | --- | --- | --- | --- | --- | --- | --- |
|  | Group 1 (*n* = 456) | Group 2 (*n* = 108) | Z | Between-group p-value | Group 1 (*n* = 456) | Group 2 (*n* = 108) | Z | Between-group p-value |
| T_tension_ | 4.46 ± 3.18 | 6.52 ± 4.06 | 4.960 | < 0.001 | 3.79 ± 3.14 | 7.08 ± 4.31 | 7.755 | < 0.001 |
| T_anger_ | 2.66 ± 2.98 | 4.27 ± 4.16 | 3.850 | < 0.001 | 2.67 ± 3.07 | 6.22 ± 5.94 | 6.747 | < 0.001 |
| T_fatigue_ | 4.94 ± 3.58 | 7.38 ± 4.34 | 5.410 | < 0.001 | 3.12 ± 3.04 | 7.74 ± 5.00 | 9.356 | < 0.001 |
| T_depression_ | 2.86 ± 3.41 | 4.92 ± 4.18 | 5.267 | < 0.001 | 2.01 ± 2.75 | 5.12 ± 5.28 | 7.178 | < 0.001 |
| T_vigour_* | 13.21 ± 4.59 | 11.09 ± 4.68 | –4.277 | < 0.001 | 15.44 ± 5.42 | 11.44 ± 5.10 | –6.773 | < 0.001 |
| T_confusion_ | 3.12 ± 2.72 | 4.84 ± 2.89 | 5.724 | < 0.001 | 2.42 ± 2.57 | 4.77 ± 3.89 | 6.150 | < 0.001 |
| T_esteem_* | 9.18 ± 3.36 | 8.36 ± 3.15 | –2.116 | 0.034 | 11.04 ± 3.67 | 9.07 ± 3.67 | –4.800 | < 0.001 |
| TMD | 95.65 ± 17.89 | 108.47 ± 21.88 | 5.528 | < 0.001 | 87.52 ± 17.63 | 110.42 ± 25.38 | 8.661 | < 0.001 |

Group 1 (non-fatigue group): RPE after BCT < 13. Group 2 (fatigue group): RPE after BCT ≥ 13. Data were shown as the mean ± standard deviation. Between-group *P* value was calculated by Mann-Whitney U test since Kolmogorov-Smirnov test verified they were non-normality data. BCT: basic combat training; POMS: Profile of Mood States Questionnaire. T_tension_, T_anger_, T_fatigue_, T_depression_, T_vigour_, T_confusion_, and T_esteem_ were the evaluation scores of 7 factors of POMS (T score). T score denoted with * were positive mood, others were negative mood. TMD: total mood disturbance evaluated by POMS.
